# Multiphasic analysis of the temporal development of the distal gut microbiota in patients following ileal pouch anal anastomosis

**DOI:** 10.1186/2049-2618-1-9

**Published:** 2013-03-04

**Authors:** Vincent B Young, Laura H Raffals, Susan M Huse, Marius Vital, Dongjuan Dai, Patrick D Schloss, Jennifer M Brulc, Dionysios A Antonopoulos, Rose L Arrieta, John H Kwon, K Gautham Reddy, Nathaniel A Hubert, Sharon L Grim, Joseph H Vineis, Sushila Dalal, Hilary G Morrison, A Murat Eren, Folker Meyer, Thomas M Schmidt, James M Tiedje, Eugene B Chang, Mitchell L Sogin

**Affiliations:** 1 Department of Internal Medicine, Division of Infectious Diseases, Ann Arbor, MI, USA; 2 Department of Microbiology and Immunology, University of Michigan Medical School, Ann Arbor, MI, USA; 3 Department of Internal Medicine, Division of Gastroenterology and Hepatology, Mayo Clinic, Rochester, MN, USA; 4 Josephine Bay Paul Center, Marine Biological Laboratory, Woods Hole, MA, USA; 5 Center for Microbial Ecology, Michigan State University, East Lansing, MI, USA; 6 Department of Microbiology and Molecular Genetics, Michigan State University, East Lansing, MI, USA; 7 Institute for Genomics and Systems Biology, Argonne National Laboratory, Argonne, IL, USA; 8 Department of Medicine, Section of Gastroenterology, The University of Chicago, Knapp Center for Biomedical Discovery, Chicago, IL, USA

**Keywords:** Pouchitis, Microbiome, Microbial ecology, Inflammatory bowel disease

## Abstract

**Background:**

The indigenous gut microbiota are thought to play a crucial role in the development and maintenance of the abnormal inflammatory responses that are the hallmark of inflammatory bowel disease. Direct tests of the role of the gut microbiome in these disorders are typically limited by the fact that sampling of the microbiota generally occurs once disease has become manifest. This limitation could potentially be circumvented by studying patients who undergo total proctocolectomy with ileal pouch anal anastomosis (IPAA) for the definitive treatment of ulcerative colitis. A subset of patients who undergo IPAA develops an inflammatory condition known as pouchitis, which is thought to mirror the pathogenesis of ulcerative colitis. Following the development of the microbiome of the pouch would allow characterization of the microbial community that predates the development of overt disease.

**Results:**

We monitored the development of the pouch microbiota in four patients who underwent IPAA. Mucosal and luminal samples were obtained prior to takedown of the diverting ileostomy and compared to samples obtained 2, 4 and 8 weeks after intestinal continuity had been restored. Through the combined analysis of 16S rRNA-encoding gene amplicons, targeted 16S amplification and microbial cultivation, we observed major changes in structure and function of the pouch microbiota following ileostomy. There is a relative increase in anaerobic microorganisms with the capacity for fermentation of complex carbohydrates, which corresponds to the physical stasis of intestinal contents in the ileal pouch. Compared to the microbiome structure encountered in the colonic mucosa of healthy individuals, the pouch microbial community in three of the four individuals was quite distinct. In the fourth patient, a community that was much like that seen in a healthy colon was established, and this patient also had the most benign clinical course of the four patients, without the development of pouchitis 2 years after IPAA.

**Conclusions:**

The microbiota that inhabit the ileal-anal pouch of patients who undergo IPAA for treatment of ulcerative colitis demonstrate significant structural and functional changes related to the restoration of fecal flow. Our preliminary results suggest once the pouch has assumed the physiologic role previously played by the intact colon, the precise structure and function of the pouch microbiome, relative to a normal colonic microbiota, will determine if there is establishment of a stable, healthy mucosal environment or the reinitiation of the pathogenic cascade that results in intestinal inflammation.

## Background

The inflammatory bowel diseases (IBD) are a heterogeneous group of chronic, relapsing ailments of unknown origin that afflict over a million people in the US. There are two major forms of IBD. Crohn’s disease is an inflammatory disorder that can involve any part of the gastrointestinal tract, but more often involves the ileo-cecal region. Ulcerative colitis (UC), on the other hand, only involves the colon and, almost without exception, extends from the rectum proximally in a continuous manner. The singular localization of these diseases suggests that topical or regional factors are important in their development. In this regard, indigenous enteric microbes may play an important role, particularly as they have been strongly implicated in the etiopathogenesis of inflammatory bowel diseases [1-4].

The search for typical bacterial pathogens for the etiology of IBD has not been particularly fruitful. More recently, the concept that specific communities of microbes play a key role in the pathogenesis of IBD has been aided by the use of culture-independent surveys of microbial community structure [5]. A growing number of studies have demonstrated that patients with IBD often have altered communities of enteric microbes or “dysbiosis” [6-8]. However, a key limitation is that these studies are generally cross sectional in design, and while associations between IBD and dysbiosis may be robust, the case for actual causation is often much weaker.

Pouchitis is an inflammatory condition of the surgically created pseudorectum reservoir that develops within 1 year in over half of UC patients who undergo total colectomy with ileal pouch anal anastomosis (IPAA) [9,10]. The condition is relatively unique to UC, as non-IBD patients (e.g., those with familial adenomatous polyposis) who undergo the same surgical procedure rarely develop pouchitis [11]. Temporal analysis of the microbiome in patients who undergo IPAA can be accomplished in a manageable timeframe with few confounding variables, and patients can serve as their own controls. These unique characteristics of IPAA in the setting of UC permit longitudinal monitoring of the microbes in the gut prior to the development of disease in a setting with a relatively high incidence of pathology.

In this study, we monitored the development of the pouch microbiota in a group of UC patients who underwent colectomy with IPAA. Starting from a time just prior to the takedown of the diverting ileostomy, we noted the transition of the pouch microbial community between multiple structural states, many of which are still distinct from the structural and functional characteristics of a healthy colonic microbiota. The driver for these changes appears to be the restoration of the flow of intestinal contents to the pouch and of the conversion of the distal ileum to a reservoir where fecal stasis occurs. It remains to be determined if the ultimate maturation of the pouch microbiome to one that resembles that seen in the healthy colon prevents the eventual development of pouchitis.

## Methods

### Study design and patients

**Figure 1:**
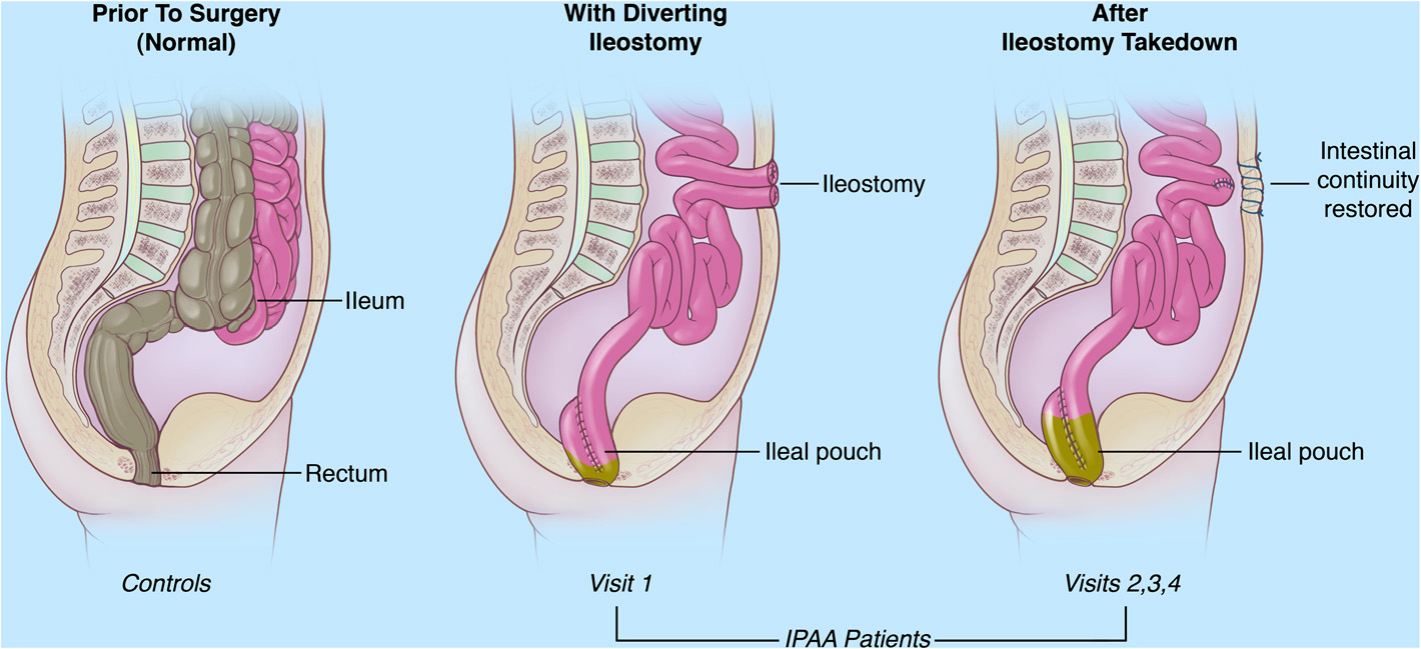
**Anatomy of ileal pouch anal anastomosis (IPAA).** Patients who undergo a two-stage IPAA procedure initially undergo a total colectomy with the construction of the ileal pouch, which is anastomosed to the rectum. Diversion of the fecal stream occurs through an ileostomy. Study subjects were initially sampled at this stage (visit 1) with specimens harvested from the diverted pouch. In the second stage of the IPAA procedure, the diverting ileostomy is taken down and continuity is restored to the ileum, restoring the flow of intestinal contents to the ileal pouch. The subsequent samples were obtained from the ileal pouch, which was accessed via the rectum (visits 2, 3 and 4). Control samples were obtained from healthy individuals who had the same anatomy as the IPAA subjects prior to the first stage of the procedure.

In this study, four patients (Table 1) with a history of UC undergoing total abdominal colectomy with IPAA (Figure 1) were identified from the outpatient and inpatient practices of gastroenterologists and colorectal surgeons at the University of Chicago Medical Center between 2010 and 2011. All four patients had a confirmed diagnosis of ulcerative colitis based on endoscopy and pathology findings, were scheduled for a total proctocolectomy with ileal pouch anal anastomosis, and were willing and able to participate in the study. Exclusion criteria included pregnancy or inability to give informed consent. All patients gave written informed consent before screening. The Institutional Review Board of the University of Chicago Medical Center approved this study protocol. Mucosal biopsy samples were also collected from the colons of healthy individuals to serve as a comparison group (Table 1). These samples were obtained without prior bowel preparation to ensure that the microbiota were not altered by this procedure. Healthy controls were otherwise healthy, and colonoscopies were performed solely for research purposes. Healthy subject 304 was on oral contraceptives at the time of colonoscopy. The other healthy controls were on no medication at the time of colonoscopy.

**Table 1:** Patient demographics

**Patient**	**Age (y)**	**Sex**	**Disease duration at colectomy (years)**	**Reason for colectomy**	**Extent of colonic inflammation**	**Tobacco use**	**Family history of IBD**
200	23	Male	2	Medically refractory	Extensive	Never	No
206	19	Male	1	Medically refractory	Extensive	Never	No
207	31	Male	2	Fulminant	Extensive	Ex-smoker	No
210	45	Male	<1	Medically refractory	Extensive	Ex-smoker	No
300	29	Female	Control n/a	n/a	n/a	Never	No
302	27	Female	Control n/a	n/a	n/a	Never	No
303	23	Male	Control n/a	n/a	n/a	Never	No
304	25	Female	Control n/a	n/a	n/a	Never	No
305	25	Male	Control n/a	n/a	n/a	Current	No

The study design was as follows. The date of ileostomy takedown was considered as the reference point for comparison of subsequent samples. One to 2 weeks prior to takedown of the patient’s loop ileostomy, a pouchoscopy was performed (visit 1; Figure 1). Mucosal biopsies and brushings of the ileal pouch were obtained, and a stool aspirate was collected. Two weeks, 4 weeks, and 2 months (visits 2, 3, 4; Figure 1) after the takedown of the ileostomy, pouchoscopy was performed with collection of mucosal biopsies, mucosal brushings and stool aspirate. The Pouch Disease Activity Index (PDAI [12]) was calculated at each visit.

Mucosal biopsies using standard biopsy forceps were obtained from the sigmoid healthy control patients, throughout the colon and from the ileal pouch in patients with ileal pouches. Mucosal brushings were also obtained in our study population (patients with ileal pouches). Cytology brushes were advanced through the endoscope, and mucosal brushings were obtained with the intent to cover a large surface area with each brush. The cytology brush was then covered in a sterile sheath prior to being removed from the endoscope. Patient 200 was on 5 mg of prednisone for 1 week following ostomy takedown. Patient 210 was on a low dose of prednisone following ostomy takedown (5 mg daily), which was slowly tapered off by visit 4. The other two patients were on no medications during the study visits.

### Sample collection and processing

Biopsy and brush samples were collected during the endoscopy, stored accordingly depending upon the downstream workflow, placed on dry ice at the time of collection (apart from those targeted for histology) and archived at −80°C thereafter. Storage conditions according to workflow were as follows: biopsies targeted for bulk DNA extraction were placed in Fecal Dry Bead Tubes (MO BIO Laboratories, Inc.), and brushes targeted for microbial cultivation were stored in cryovials containing 1× anaerobic GBSS buffer (supplement material) supplemented with DMSO (5% final concentration). Bulk DNA was extracted from biopsies using a modified protocol for the Roche MagNA Pure System incorporating mechanical disruption (bead beating) as described previously [13].

### Amplicon library construction

Replicate amplicon libraries for bacterial 16S v3 through v5 regions (Bv3v5) were constructed from all samples. The adapter and 16S rRNA-encoding gene-specific sequences are shown in Table 2. Each primer contains either the A or B 454 Titanium amplicon adapter followed by a 5-nt multiplex identifier (MID; barcode) and ends with the 16S specific sequence. Bv3v5 amplicons were generated using a pool of two forward and three reverse primers, and the MID is present in all five oligonucleotides. All MIDs differ by at least two bases and contain no homopolymers.

**Table 2:** Primers for amplification of variable regions of the 16S rRNA-encoding gene

**Primer domain**	**Sequence, 5**^**′ **^**to 3**^**′**^
“A” adapter	CGTATCGCCTCCCTCGCGCCATCAG
“B” adapter	CTATGCGCCTTGCCAGCCCGCTCAG
341 F2	CCTACGGGNGGCWGCAG
341 F3	TCTACGGAAGGCTGCAG
926R1	CCGTCAATTCNTTTRAGT
926R3	CCGTCAATTTCTTTGAGT
926R4	CCGTCTATTCCTTTGANT

Both primer sets were designed to capture over 95% of known eubacterial diversity. They match 16S genes from 50 to 100% of the members of all known phyla in our reference database based on the SILVA 106 release [14] with the exception of a few small phyla such as OP11 and SR1, which were not expected to be encountered in gut communities.

The individual oligos were mixed in equal proportions to create F/R primer pools. 16S rRNA-encoding gene amplicons were generated by polymerase chain reaction containing 1X Platinum HiFi Taq polymerase buffer, 1.6 units Platinum HiFi polymerase (Invitrogen/LifeTechnologies), 3.7 mM MgSO_4_, 200 uM dNTPs (PurePeak polymerization mix, Pierce/ThermoFisher) and 50 nM combined primers. Between 5–25 ng of sample DNA was added to a master mix to a final volume of 100 ul, and this was divided into three replicate 33-ul reactions. Cycling conditions included an initial denaturation at 94°C for 3 min; 30 cycles of 94°C for 30 s; 60°C for 45 s, 72°C for 1 min; and a final extension at 72°C for 2 min using an Applied Biosystems 2720 or 9700 cycler. The three replicates were pooled and analyzed on a Bioanalyzer DNA1000 chip. Reactions were cleaned and products under 300 base pairs removed using Ampure beads at 0.75× volume (Agilent Technologies). The final products were resuspended in 100 ul of 10 mM Tris-EDTA, quantitated using PicoGreen Quant-IT assay (Invitrogen/LifeTechnologies) and assayed again on a DNA1000 chip.

### 454 Sequencing and data analysis

Up to 40 amplicon libraries were pooled prior to emulsion PCR. The emPCR, enrichment and sequencing were done according to current Roche Titanium amplicon sequencing protocols (Lib-A emPCR reagents, XLR sequencing reagents, two region PicoTitre plate). A typical sequencing run generated an average of 80,000 tags per sample (Additional file 1: Table S1). Image processing and signal calling are done using the Roche amplicon processing pipeline (version 2.5.3) with recursive phase correction algorithm.

Pyrosequencing reads were quality-filtered by removing reads that did not have exact matches to the MID and the proximal primer (341 F); that contained an ambiguous base (N); that lacked a conserved distal anchor region used for trimming; or that had an average quality score less than 30 [15]. The v3v5 anchor is 5^′^-GGATTAGNTACCC-3 (position 785 F in *E. coli).* Reads were trimmed after anchor sequence. Chimeras were removed using UChime [16], combining both the *de novo* and reference database (ChimeraSlayer GOLD) modes. Taxonomy was assigned using GAST [17] and the data uploaded to the Visualization and Analysis of Microbial Population Structures website (VAMPS: http://vamps.mbl.edu) for analysis. OTU clustering was performed using UClust [18] using 97% sequence identity. All trimmed and filtered sequence tag data and taxonomy are available on VAMPS under the project name prefixes HMP_200, HMP_206, HMP_207, HMP_210 and HMP_300 and have been submitted to NCBI’s sequence read archive (SRA) with the project ID http://www.ncbi.nlm.nih.gov/bioproject/46315. The 16S PCoA analyses were performed using the Morisita-Horn, Yue-Clayton and UniFrac inter-community distance metrics at both full sampling depth and with each data set subsampled to 8,461 sequences (the smallest data set size). All PCoAs were virtually identical.

### Cultivation from brush samples

Brush samples were frozen in 1 ml of GBSS (glucose-free buffered salt solution, recipe at http://microbiomes.msu.edu/resources/) buffer (with 5% dimethyl sulfoxide) until processing. Samples were thawed on ice and vortexed for 30 s. The brush was removed and vortexed for 30 s in a second tube with GBSS buffer, then the brush was discarded. The suspended samples were combined, homogenized by vortexing for an additional 60 s before aliquots were removed for microscopy and cultivation.

Viable cell counts were obtained through plating of serial dilutions. Triplicate dilution series for each sample (10-fold for anoxic cultivation, 4-fold for oxic cultivation) were performed in GBSS buffer on ice in the same environment used for downstream cultivation, followed by plating onto complex media containing various carbon, nitrogen and sulfate sources common in the human GI tract (http://microbiomes.msu.edu/resources/). Plates were incubated at 37°C for as many as 7 days in anoxic (2–3% H_2_, 5% CO_2_, N_2_) or oxic (ambient, 21% O_2_) atmospheres. The total number of colony-forming units (CFUs) at day 5 was used to compare viable cell density (CFU/ml) for all samples.

To determine the overall density of microorganisms present in the sample, direct cell counts were performed. Samples were fixed in 3.7% formaldehyde overnight at 4°C and treated with DNase I (0.5 U/μl, Roche, Indianapolis, IN) at 37°C for 1 h, followed by mixing with 0.1 M hydrogen chloride (1:1, v/v). Further dilutions were made as necessary in 0.1 M hydrogen chloride; 4 μl of the treated sample was loaded into the counting chamber (Hawksley Helber Bacteria 1 Cell Thoma, Sussex, UK). Microbes deposited in nine grids (200 μm × 200 μm/grid) were counted for each of triplicate samples load. The average number of cells was used to estimate total cell counts in the original sample. Repeated measures ANOVA was performed to test the statistical significance of changes in direct or viable cell counts over sampling time points. A linear regression model was applied for log-transformed direct cell counts to estimate microbial population doubling time.

### Screening for butyrate producing taxa

The 16S data were harvested for known butyrate producers of the human colon (based on [19]) at the genus level, except for the functionally diverse *Clostridia* where species discrimination was applied (Additional file 1: Table S2). Additional butyrate-producing candidates were revealed in luminal aspirate samples by the functional gene targeting approach presented in the companion manuscript Vital et al. [20] and were included in the analysis. All results were normalized to five *16S rRNA*-encoding gene copy numbers, which represent the average for *Firmicutes* and *Bacteroidetes*, the two most abundant phyla in the gut. Candidates were divided into two groups based on whether they have butyrate kinase (*buk*) or butyryl-CoA:acetate CoA-transferase (*but*) genes, the final genes of the two main microbial butyrate synthesis pathways, to facilitate data comparison with the results from the companion manuscript.

## Results

### Temporal shifts in the pouch microbiome following ileostomy takedown

**Figure 2:**
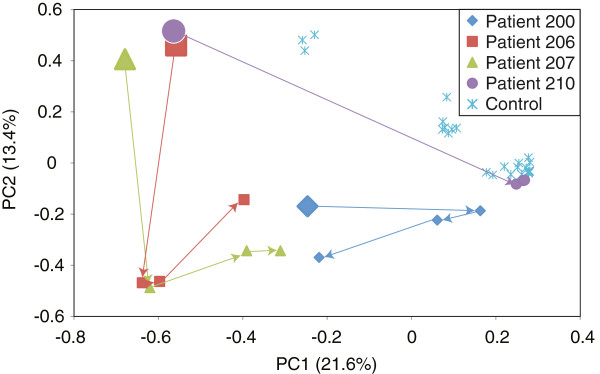
**Shift in mucosal microbiota of the pouch after takedown of diverting ileostomy in patients who had undergone ileal pouch anal anastomosis visualized by principle coordinates analysis of the Unifrac metric based on V3-5 16S rRNA-encoding gene amplicons.** The initial time point, prior to takedown of the ileostomy, is indicated by the larger symbols and subsequent time points at the end of the arrows. For comparison, colon mucosal biopsies from healthy individuals are included on the ordination.

**Figure 3:**
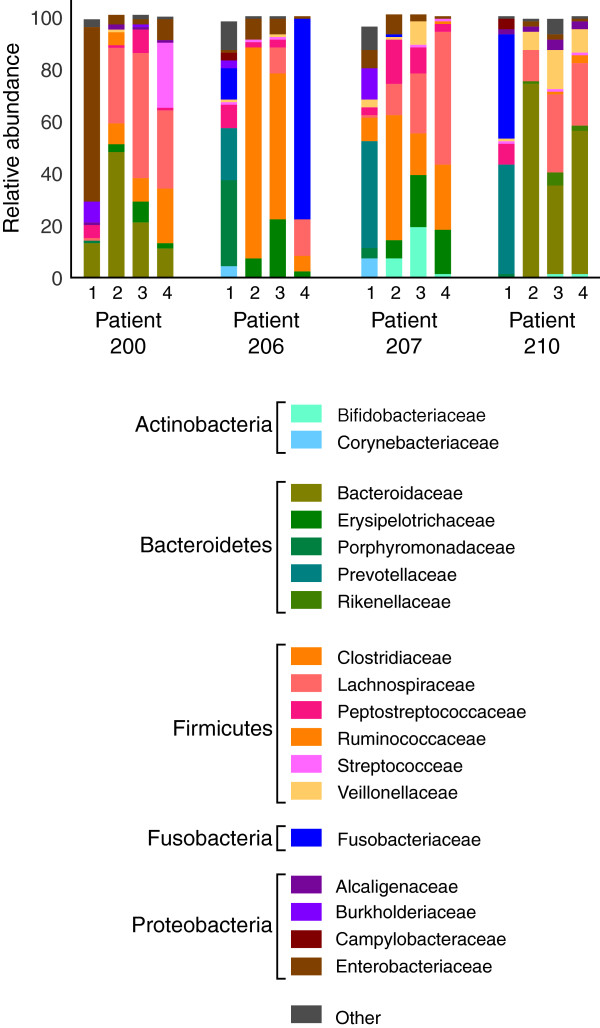
**Taxonomic classification of V3-5 16S rRNA-encoding gene amplicon sequences from patients who had undergone ileal pouch anal anastomosis.** The relative abundance of major phylotypes, classified to the level of family and grouped by phylum, is indicated. Time point 1 is prior to ileostomy takedown, and time points 2, 3 and 4 are 2, 4 and 8 weeks after takedown.

Changes in the pouch mucosal microbiota after restoration of the fecal stream with the ileal pouch were monitored by sampling before and after takedown of the diverting ileostomy (Figure 1). Amplicons targeting the V4-6 region of the 16S rRNA-encoding gene were generated and subjected to multiplex pyrosequencing. Analysis of the 16S pyrotag data by ordination revealed that ileostomy takedown was associated with a shift in the composition of the pouch mucosal bacterial community. In all four patients the most dramatic change in community structure was seen comparing the sample obtained prior to ileostomy takedown to the samples following restorative surgery (Figure 2). Less variation was seen in the community structure among the three samples obtained after ileostomy takedown. In accordance with previous studies of the gut microbiota, each subject possessed a unique community of organisms when the pyrotags were classified at all taxonomic levels (phylum to genus) (see Figure 3, Additional file 1: Table S3 for family level taxonomy, other levels not shown). At the phylum level, the majority of the pyrotags were affiliated with members of the Firmicutes and Bacteroidetes, in particular after the ileostomy takedown.

As a comparison, we obtained samples from the colonic mucosa of healthy subjects without inflammatory bowel disease via colonoscopy. When these samples were subjected to 16S pyrotag analysis and compared to the pouch mucosa samples, three of the four samples obtained prior to ileostomy takedown clustered as one group, one patient clustered alone, and samples from normal colonic mucosa clustered separately (Figure 2). The samples obtained from the pouch following ileostomy takedown appeared to show an intermediate community structure, although one subject (patient 210) developed a community that fell within the community structures encompassed by the normal colonic samples.

**Figure 4:**
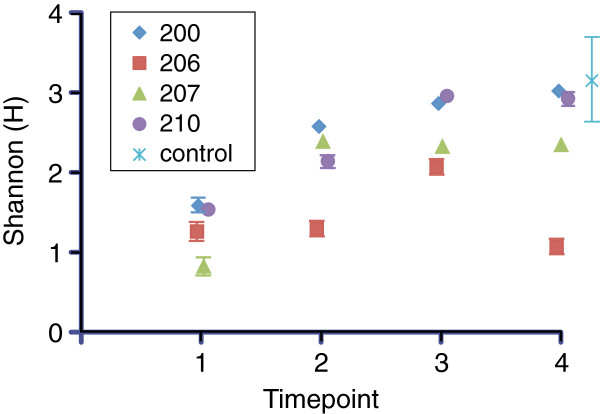
**Change in diversity of the mucosal microbial communities inhabiting the ileal/anal pouch of patients after ileostomy takedown.** The Shannon diversity index was calculated for each community based on OTUs defined at sequence divergence ≥ 0.03.

We compared the overall diversity of the mucosal communities at each time point by calculating the Shannon index (Figure 4). For three of the subjects, there was an increase in overall diversity following ileostomy takedown, but in the fourth subject (patient 206), this increase was not seen, although the community composition did change substantially. By the time the third and fourth samples were taken, for two of the patients (200 and 210), the overall diversity was in the range of that seen in the control colon mucosa samples.

### Cultivation of mucosal samples demonstrates a shift from facultative to obligate anaerobes following ileostomy takedown

**Figure 5:**
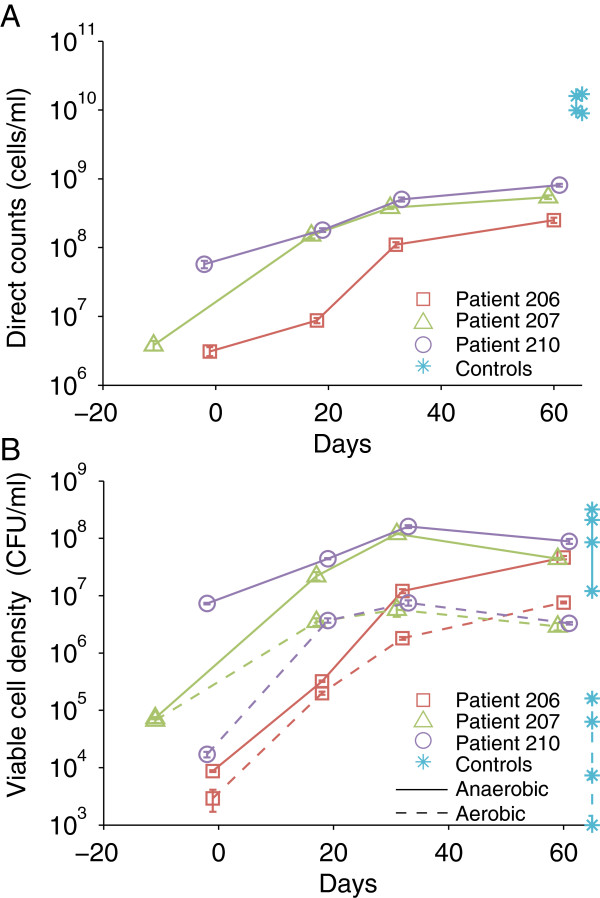
**Changes in the direct counts and viable cell density from pouch mucosal samples.** Brush samples obtained during endoscopy were loaded onto a counting chamber for direct cell counts (**A**). Error bars were standard errors of three counts per sample. Anaerobic and aerobic cultivation on complex medium was performed. The number of colony-forming units (CFU) on day 5 was used to calculate viable cell density (CFU/ml) (**B**). Error bars were standard errors of CFU number on triplicate agar plates from independent serial dilutions. For comparison, colon mucosal brushes/biopsies from healthy individuals (control) are included in the figure.

We also tracked the development of pouch microbiota by direct and viable cell counts. The overall density of microorganisms in the pouch mucosa continuously increased by approximately two orders of magnitude during the sampling time series (Figure 5, *p* = 0.003). More interestingly, the increase was modeled as exponential (adjusted R^2^ = 0.73-0.88) over the whole sampling period, suggesting an active proliferation of the microbial community after ileostomy takedown. Model regression led to an estimated doubling time of 9 to 16 days for the pouch microbiota over the sampling time. By the last sampling point, which was 2 months after ileostomy takedown, the overall microbial cell density in all three patients was still at least one order of magnitude lower than that in healthy colon mucosa samples.

Mucosal samples obtained with a cytology brush were cultured under anoxic and oxic atmospheres to examine the response of the microbial communities to oxygen. The numbers of both anaerobic and aerobic cultivars increases rapidly after ileostomy takedown and then declines gradually 4 weeks later. The anaerobic counts are always higher than the aerobic counts. More interestingly, the ratio of the two viable counts (shown as the vertical distance between anaerobic and aerobic counts at the same time point in Figure 5) increased from 1.6 to more than 20 over time following ileostomy takedown. It suggests a shift from facultative to more obligate anaerobes of the pouch microbiota. In contrast, healthy colons had similar levels of anaerobic counts, but two to three orders of magnitude lower aerobic cell densities compared to the pouch samples.

### Shifts in diversity correlated with an increase in potential butyrate metabolism following ileostomy takedown

**Figure 6:**
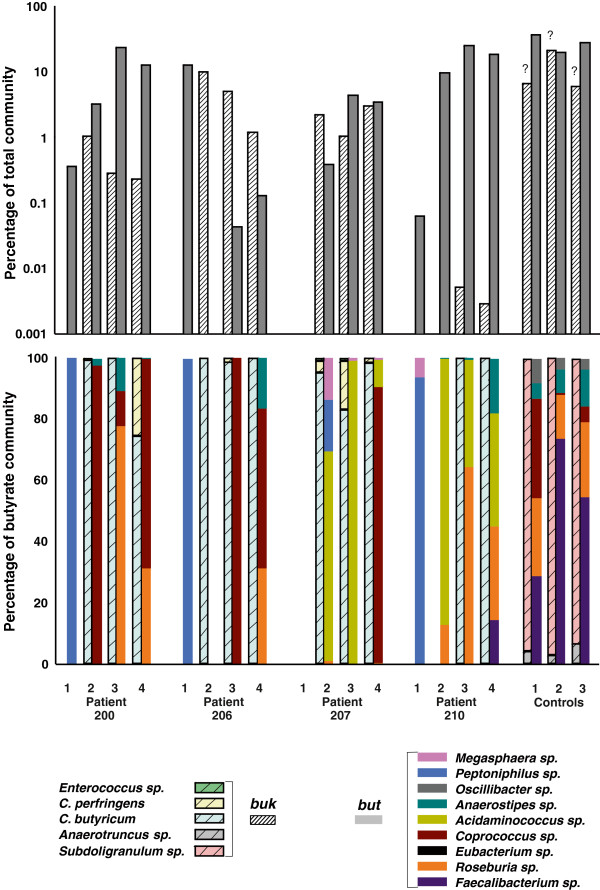
**Analysis of 16S rRNA-encoding gene data for butyrate-producing communities.** Panel **A** shows the cumulative result of obtained candidates associated with butyryl-CoA:acetate CoA-transferase (but; grey bar) and butyrate kinase (buk; white stripped bar). Below (**B**) individual compositions of communities are given. Results are corrected for multiple 16S rRNA-encoding gene copy numbers for individual bacteria. ? = butyrate production was shown for one strain of Subdoligranulum sp. as it is unclear whether all members of this genus have the potential to synthesize butyrate.

The 16S pyrotag data and the cultivation data document significant changes in the community inhabiting the pouch mucosa following ileostomy takedown. To determine if these changes in community structure were associated with potential functional changes, we analyzed the 16S rRNA gene data from biopsy samples at the genus and species level for butyrate-producing candidates (Figure 6). Few potential butyrate-producing bacteria were detected at the time of the initial visit (visit 1; except for patient 206, who exhibited a considerable *Peptoniphilus* community), whereas abundant communities with this potential were established in all patients shortly after ileostomy takedown (visit 2) and remained consistent during the remaining two sampling points. Profiles were distinct, however, between individuals with abundant *buk-*carrying candidates (mainly *Clostridium butyricum* and *C. perfringens*) in patients 206 and 207, whereas species associated with *but* were dominant in patients 200 and 210. Only patient 210 exhibited both *Faecalibacterium sp.* and *Roseburia sp.,* which are considered to be among the most abundant butyrate producers in the healthy colon [19] (as also observed in our control samples). Additional *but-*linked bacteria, namely *Acidaminococcus* sp. and *Coprococcus* sp., were present at high concentrations in several samples.

## Discussion

In this study we describe the establishment of the enteric microbial community in the ileal pouch of patients with a history of UC who have undergone total colectomy with IPAA. Our study is unique in examining the relationship of the microbiota and pouchitis and offers several advantages: (1) a clearly defined starting point when all patients are free of disease and off medications, (2) the opportunity to observe the development of a nascent pouch microbiota relative to the maturation of the ileal pouch, (3) sampling of mucosa-associated pouch microbiota without colonic lavage [21] and (4) the identification of pouch microbial communities that may be associated with health or increased risk of disease.

We were able to determine that in all four patients the pouch microbial community evolved over time, but it did so in a manner that was unique to each individual. As expected, the shift in the microbial community in each individual was most evident immediately after closure of the diverting ileostomy and reinstitution of the fecal stream through the pouch. While the microbial communities continued to evolve over time, the changes were less pronounced. The microbial community appeared to move toward a more “healthy” colonic community at each subsequent visit in patients 206, 207 and 210. However, in patient 200, while the microbial community initially moved toward a “healthy” colonic community, it drifted away from the healthy colonic community structure 1 and 2 months after ileostomy closure. Interestingly, this patient whose microbial community was less similar over time to the communities of the healthy colons developed chronic pouchitis 7 months after visit 4 and was diagnosed with *C. difficile* of the ileal pouch 10 months following visit 4. Patients 206 and 207 both had episodes of acute pouchitis at 16 and 17 months, respectively, following closure of the ileostomy. Both patients responded well to antibiotic treatment. Patient 210’s microbial community structure moved closest to the community structure observed in healthy colon samples, and this patient has had the best clinical outcome of the four subjects. Two years into the study, he continues to do well with no episodes of pouchitis.

Although our sample size was small, our findings suggest that the failure to develop a mature microbial community similar to that found in the healthy colon in the months following ostomy closure increases susceptibility to pouchitis. Alternatively, if an individual’s pouch microbiota develop into a diverse, mature community similar to that found in the healthy colon, we propose the risk of pouchitis is decreased. Falk and colleagues also performed a longitudinal study in two patients with a history of UC undergoing colectomy with IPAA [22]. They followed these two patients for 1 year after stoma closure. Although the microbial profiles were quite different between the two patients, both individuals’ mucosal-associated microbiota evolved over time and became more “colon-like.” Neither patient developed pouchitis.

Others have compared the pouch microbiota in patients with and without pouchitis, although the results from these various studies have varied, and no clear association between particular bacterial profiles has been identified [23-26]. Komanduri et al. pooled mucosal samples from patients with UC healthy pouch, UC pouchitis and non-IBD controls. They described shifts in the microflora (dysbiosis) in patients with pouchitis, with an increase in the proportion of *Fusobacter* (phylum Proteobacteria) and a decrease in *Streptococci* (phylum Firmicutes). In another study examining the mucosal-associated microbiota from ileal pouches, diversity was greater in the UC patients without pouchitis compared to the UC patients with pouchitis. There were no specific phylotypes associated with pouchitis [23].

Dysbiosis of the intestinal microbiota is commonly found in patients with IBD [27], but in almost all cases, the question remains as to whether these changes are causes or consequences of the activated immune and inflammatory condition. Typically, large changes in 16S rRNA gene-based profiles are observed at the phylum level, characterized by blooms of Proteobacteria and changes in the relative abundance of Firmicutes and Bacteroidetes [28]. Significant differences in the functional metagenomes of enteric microbes from healthy and IBD patients have also been shown [29]. However, the observed structural and functional changes are not unique to human IBD and can be found in other non-IBD inflammatory states [30,31] and in experimental colitis [32-34], suggesting that these changes are largely a consequence of the altered immune and inflammatory state. These large, descriptive data sets have shed little light on fundamental mechanisms of IBD etiopathogenesis and are often confounded by difficult to control variables inherent to clinical studies, including differences in individual microbiomes, medication (including antibiotics), diet, genetics, and environmental and lifestyle factors.

Several studies have suggested that the overall diversity of the gut microbiota is decreased in the setting of IBD. However, our results indicate that diversity in and of itself (as measured by a metric such as the Shannon diversity) is not necessarily a predictor of disease. While it is true that patients 210 and 200 both developed pouch mucosal communities that were similar in overall diversity to that seen in the normal colon, patient 200 developed severe, refractory pouchitis, while patient 210 remains healthy after 2 years of follow-up. Rather than using overall diversity as a measure of “health” of the microbiome, it is likely that the specific composition and thus function of the community are the key predictors. In this regard, the community in patient 210 is most similar in composition and structure to that seen in the healthy colon, while the community in patient 200 is quite distinct, despite having relatively high overall diversity. This is also reflected in the relative potential of the communities to produce the beneficial SCFA butyrate where the community in patient 210 had a much higher potential of butyrogenesis compared to that in patient 200.

The importance of specific microbial functions is further indicated in our analysis of the butyrogenic potential of an individual patient’s microbiome. Abundant butyrate-producing communities were established in all patients after ileostomy takedown, but only patient 210 exhibited both taxa *Roseburia* sp. and *Faecalibacterium* sp. (Figure 6), which are considered to be the main butyrate producers in healthy colons [19,20]. In our companion work (Microbiome, submitted in parallel to this manuscript), we specifically investigated the diversity (via a pyrosequencing strategy) and abundance (via quantitative PCR) of butyrate-producing genes in corresponding luminal samples taken at the same time as mucosa samples. All patients established an abundant butyrate-producing community (approximately 5–26% of the total community) after ileostomy takedown, but with distinct profiles between patients, where patient 210 was the only individual exhibiting a *but/k* profile similar to those of the control samples. Whereas the overall patterns between 16S rRNA gene analysis (of luminal samples) and the functional gene-targeted approach were consistent, only the latter could reveal butyrate-producing gene families and their inferred taxa in detail to more directly evaluate the butyrogenic potential. Unfortunately, it was not possible to use the functional gene-targeted approach in this mucosal study because of the low amount of bacterial DNA in most biopsy samples. However, 16S rRNA gene patterns specific for butyrate producers from Vital et al. [20] were identical to results presented here (Figure 6), suggesting consistency in functional gene profiles between mucosa and luminal derived samples as well. Indeed, we were able to retrieve qPCR data from mucosal samples of patient 210 (only visit 3 and 4) and found *but* genes linked to *Roseburia* sp./*Eubacterium* sp. and *F. prausnitzii* (data not shown) at abundances similar to luminal aspirates and consistent with the 16S rRNA gene data presented in Figure 6. The observed progression of patient 210 toward a “healthy type” bacterial community based on global 16S rRNA analysis was consistent with the development of a specific butyrate-producing community similar to that of healthy colons. The question of how individual bacterial profiles relate to the actual production of butyrate and whether abnormal communities perform equally compared to regular ones is under study.

This study describes the establishment of the pouch microbiota in a longitudinal manner. This is the first step toward understanding how the intestinal microbiota influence or trigger the development of the inflammatory response seen in IBD. Despite our small sample size, we can confirm that there is an evolution of the microbial community in all individuals. These changes were apparent when the communities were followed by multiple methodologies. This includes the use of culture-independent surveys based on retrieval of 16S rRNA-encoding gene sequences, specific culture-based techniques and targeted analysis of functional genes.

Structurally and functionally, we find that the mucosal microbial community of the pouch moves toward a community more similar to a colonic microbial community following ileostomy takedown. Nonetheless, the membership within the community is distinct from the colonic microbial community, likely secondary to the influence of the small intestinal epithelium on the development of the pouch microbiota and the overall anatomic differences inherent to IPAA. The establishment of a pouch microbiota similar to a healthy colonic microbial community is potentially protective against pouchitis. In the future, longitudinal studies, such as this one, may provide the opportunity to identify patterns of the microbial community, both in terms of structure and function, that predict the onset of inflammation or disease in at-risk individuals.

## Conclusions

The results of this study demonstrate a marked shift in the structure and function of the microbiota that inhabit the mucosa of patients who have ileal-anal pouch anastomosis for ulcerative colitis. This shift is characterized by a transition to a community that is selected for fermentation, preferentially a beneficial one such as butyrate production. It is likely that the community structure and function of the pouch microbiome will influence the likelihood of the development of pouchitis, with the development of a pouch microbiome that resembles that seen in the normal colon being protective against the development of disease. Monitoring of the development of the pouch microbiome may be able to predict which patients require more aggressive monitoring for the development of pouchitis and potential early treatment/prevention using modalities designed to shift the microbiota into a more normal community.

### Supporting data

The data sets supporting the results of this article are available in the VAMPS repository (http://vamps.mbl.edu), which is supported by NSF grant NSF DBI-0960626 to S. Huse.

## Abbreviations

IPAA: ileal pouch anal anastomosis; IBD: inflammatory bowel diseases; UC: ulcerative colitis; PDAI: pouch disease activity index; MID: multiplex identifier; buk: butyrate kinase; but: butyryl-CoA:acetate CoA transferase

## Competing interests

The authors declare that they have no competing interests.

## Authors’ contributions

VY contributed to the study design, coordinated the various study sites and drafted the manuscript. SG and JV performed amplicon sequencing under the direction of HM. AE, PS, SH, HM and MS analyzed the 16S amplicon data and contributed to writing the manuscript. JB, NH, LR and SD were involved in the acquisition, processing and analysis of mucosal samples, and JK and GR performed the endoscopic procedures. LR also contributed to the study design and writing the manuscript. RA recruited and schedule all the patients, maintains the patient metadata and performed patient follow-up. MV and JT designed and performed the functional gene analysis and contributed to drafting the manuscript. DA contributed to the study design, set up a tracking system for coordination and distribution of samples with JB, and contributed to writing the manuscript with FM. DD cultivated and characterized bacteria from the pouch. TS oversaw the cultivation portion of the project and contributed to the writing of the manuscript. EC conceived the overall study design with VY, MS, JT, TS and FM, assisted with the writing of the manuscript and analysis of data, and created the infrastructure at U of C for this program. All authors read and approved the final manuscript.

## Supplementary Material

Additional file 1 **Counts for pyrosequencing tags in study. Table S2** Butyrate-producing candidates searched for in the 1*6S rRNA* gene data and their corresponding gene copy numbers based on rrnDB (//rrndb.mmg.msu.edu) and IMG (//img.jgi.doe.gov). **Table S3** Taxonomic composition for pouch communities at four time points (companion data for Figure 3). Click here for file
