# Supplementary material for: Multiphasic analysis of the temporal development of the distal gut microbiota in patients following ileal pouch anal anastomosis
Source: Microbiome. 2013 Mar 4;1:9. doi: 10.1186/2049-2618-1-9 (PMC3971607; doi:10.1186/2049-2618-1-9)
Supplement: Additional file 1 — Counts for pyrosequencing tags in study. Table S2 Butyrate-producing candidates searched for in the 16S rRNA gene data and their corresponding gene copy numbers based on rrnDB (//rrndb.mmg.msu.edu) and IMG (//img.jgi.doe.gov). Table S3 Taxonomic composition for pouch communities at four time points (companion data for Figure 3). [file 2049-2618-1-9-S1.doc]

Additional file 1 Table S1 Counts for pyrosequencing tags in study

| Subject | timepoint | Read counts |
| --- | --- | --- |
| 200 | 1 | 3,719 |
| 200 | 2 | 52,527 |
| 200 | 3 | 64,103 |
| 200 | 4 | 83,518 |
| 206 | 1 | 9,451 |
| 206 | 2 | 84,767 |
| 206 | 3 | 65,714 |
| 206 | 4 | 112,188 |
| 207 | 1 | 54,545 |
| 207 | 2 | 55,387 |
| 207 | 3 | 106,921 |
| 207 | 4 | 111,155 |
| 210 | 1 | 66,820 |
| 210 | 2 | 158,345 |
| 210 | 3 | 178,947 |
| 210 | 4 | 88,256 |
| 300s | NA | 521,559 |

Additional file 1 Table S2 Butyrate-producing candidates searched for in the 1*6S rRNA* gene data and their corresponding gene copy numbers based on rrnDB (http://rrndb.mmg.msu.edu) and IMG (http://img.jgi.doe.gov).

| **name** | ***16S rRNA-*encoding gene copy number** |
| --- | --- |
|  |  |
| *Acidaminococcus sp.* | 5 |
| *Anaerofustis sp.* | 2 |
| *Anaerotruncus sp.* | 4 |
| *Brachysoira sp.* | ? |
| *Butyrivibrio sp.* | 6 |
| *Clostridium acetobutylicum* | 11 |
| *C. beijerinckii* | 14 |
| *C. barlettii* | ? |
| *C. botulinum* | 9 |
| *C. butyricum* | 18 |
| *C. carboxidivorans* | 13 |
| *C. difficile* | 10 |
| *Clostridium sp. GM2/1* | ? |
| *Clostridium sp. M62/1* | 1 |
| *Clostridium sp. SS2/1* | 12 |
| *Clostridium sp. SS3/4* | ? |
| *Clostridium sp. SSC2* | 1 |
| *Clostridium sp. SY8519* | 4 |
| *Clostridium sp. 1_7_47 FAA* | 1 |
| *Clostridium sp. 7_2_43 FAA* | 1 |
| *C. perfringens* | 10 |
| *C. sporogenes* | 8 |
| *C. symbiosum* | 1 |
| *C. tetani* | 6 |
| *Coprococcus sp.* | 3 |
| *Enterococcus sp.* | 5 |
| *Eubacterium sp.* | 5 |
| *Faecalibacterium sp.* | 5 |
| *Megasphaera sp.* | 2 |
| *Oscillibacter sp.* | 3 |
| *Peptoniphilus sp.* | 1 |
| *Subdoligranulum sp.* | 1 |
| *Treponema* | 2 |

? – no data available.

Additional file 1 Table S3 Taxonomic composition for pouch communities at 4 timepoints (companion data for figure 3)

| Phylotype | 200_1 | 200_2 | 200_3 | 200_4 | 206_1 | 206_2 | 206_3 | 206_4 | 207_1 | 207_2 | 207_3 | 207_4 | 210_1 | 210_2 | 210_3 | 210_4 |
| --- | --- | --- | --- | --- | --- | --- | --- | --- | --- | --- | --- | --- | --- | --- | --- | --- |
| Clostridiaceae | 0% | 8% | 9% | 21% | 0% | 81% | 56% | 6% | 9% | 48% | 16% | 25% | 0% | 0% | 0% | 0% |
| Fusobacteriaceae | 0% | 0% | 0% | 0% | 12% | 0% | 0% | 77% | 0% | 1% | 0% | 0% | 40% | 0% | 0% | 0% |
| Bacteroidaceae | 13% | 48% | 21% | 11% | 0% | 0% | 0% | 0% | 0% | 0% | 0% | 0% | 0% | 74% | 34% | 55% |
| Enterobacteriaceae | 67% | 4% | 2% | 8% | 1% | 8% | 6% | 1% | 7% | 8% | 3% | 1% | 0% | 2% | 2% | 1% |
| Lachnospiraceae | 1% | 29% | 48% | 30% | 0% | 0% | 10% | 14% | 1% | 12% | 23% | 51% | 0% | 12% | 30% | 24% |
| Prevotellaceae | 0% | 0% | 0% | 0% | 20% | 0% | 0% | 0% | 41% | 0% | 0% | 0% | 42% | 0% | 0% | 0% |
| Porphyromonadaceae | 1% | 0% | 0% | 0% | 33% | 0% | 0% | 0% | 4% | 0% | 0% | 0% | 1% | 0% | 0% | 0% |
| Streptococceae | 0% | 0% | 0% | 25% | 1% | 1% | 1% | 0% | 0% | 0% | 1% | 1% | 1% | 0% | 1% | 1% |
| Erysipelotrichaceae | 0% | 3% | 8% | 2% | 0% | 7% | 22% | 2% | 0% | 7% | 20% | 17% | 0% | 0% | 0% | 0% |
| Bifidocateriaceae | 0% | 0% | 0% | 0% | 0% | 0% | 0% | 0% | 0% | 7% | 19% | 1% | 0% | 0% | 1% | 1% |
| Peptostreptococcaceae | 5% | 1% | 9% | 1% | 9% | 2% | 3% | 0% | 3% | 17% | 10% | 3% | 8% | 0% | 0% | 0% |
| Veillonellaceae | 0% | 1% | 0% | 0% | 1% | 0% | 1% | 0% | 3% | 1% | 9% | 0% | 1% | 7% | 15% | 9% |
| Burkholderiaceae | 8% | 0% | 1% | 0% | 3% | 0% | 0% | 0% | 12% | 0% | 0% | 0% | 0% | 0% | 0% | 0% |
| Corynebacteriaceae | 0% | 0% | 0% | 0% | 4% | 0% | 0% | 0% | 7% | 0% | 0% | 0% | 0% | 0% | 0% | 0% |
| Rikenellaceae | 0% | 0% | 0% | 0% | 0% | 0% | 0% | 0% | 0% | 0% | 0% | 0% | 0% | 1% | 5% | 2% |
| Peptococcaceae | 0% | 0% | 0% | 0% | 2% | 0% | 0% | 0% | 5% | 0% | 0% | 0% | 0% | 0% | 0% | 0% |
| Ruminococcaceae | 0% | 5% | 0% | 0% | 0% | 0% | 0% | 0% | 0% | 0% | 0% | 1% | 0% | 0% | 1% | 3% |
| Alcaligenaceae | 1% | 2% | 1% | 1% | 0% | 0% | 0% | 0% | 0% | 0% | 0% | 0% | 2% | 2% | 4% | 3% |
| Campylobacteraceae | 0% | 0% | 0% | 0% | 3% | 0% | 0% | 0% | 0% | 0% | 0% | 0% | 4% | 0% | 0% | 0% |
| Other | 3% | 1% | 2% | 1% | 11% | 1% | 1% | 0% | 9% | 0% | 0% | 0% | 1% | 1% | 6% | 1% |
